# Supplementary material for: Structure and inhibition mechanisms of Mycobacterium tuberculosis essential transporter efflux protein A
Source: Nat Commun. 2025 Apr 1;16:3139. doi: 10.1038/s41467-025-58133-6 (PMC11961569; doi:10.1038/s41467-025-58133-6)
Supplement: Supplementary file 1 — Supplementary Information [file 41467_2025_58133_MOESM1_ESM.pdf]

Supplementary information

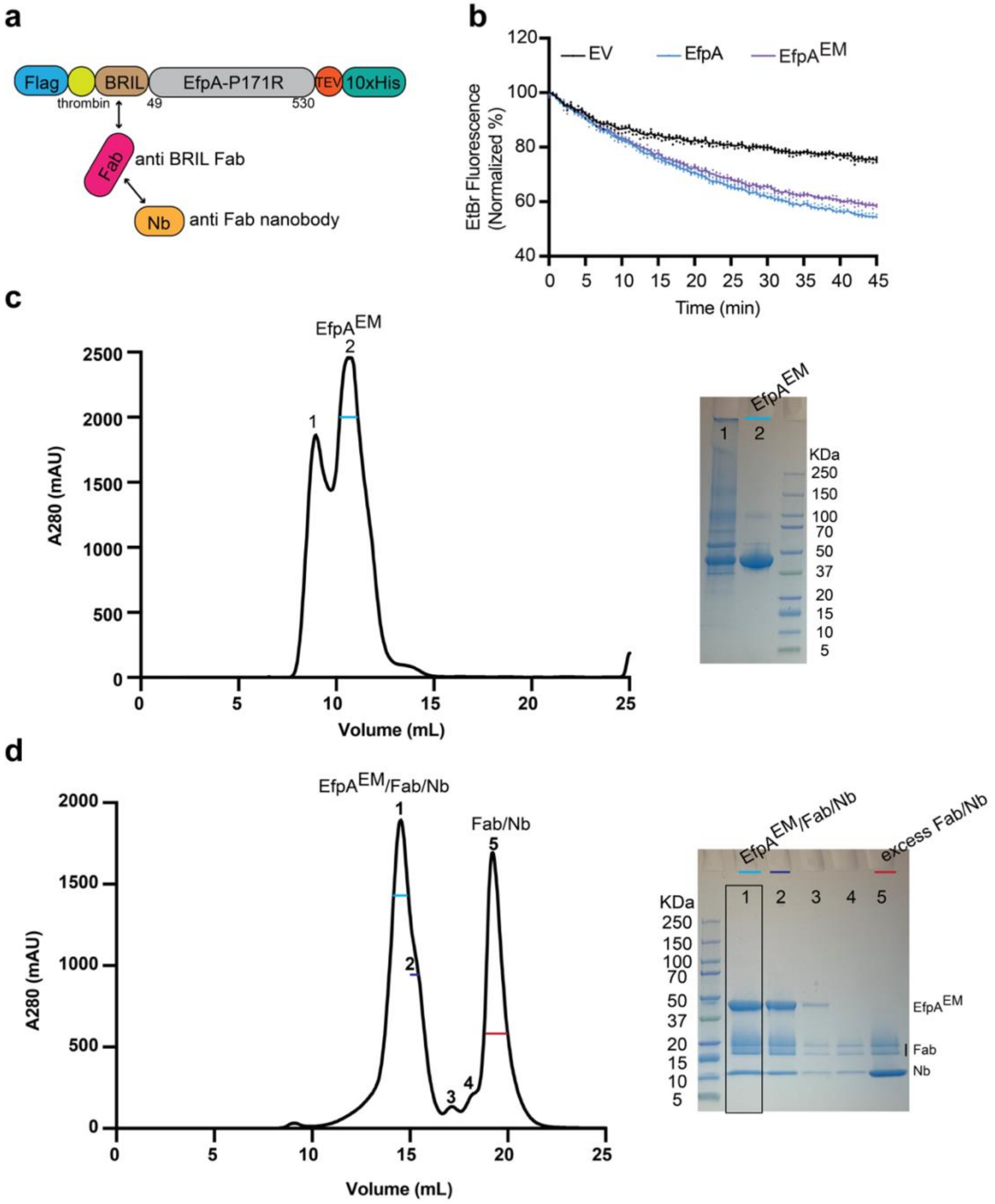

Supplementary Fig. 1 | EfpA^{EM} construct design and purification. a, Construct design and complex formation strategy for EfpA^{EM}. **b,** Cell-based transport of EtBr by EfpA. *E. coli* JD838 strain (Δ*mdfA*Δ*acrB*Δ*ydhE::kan*) expressing wildtype EfpA (light blue), EfpA^{EM} (purple) and empty vector (black) were pre-loaded with 20 μM EtBr. After stimulation with glucose, ethidium fluorescence intensity decay at 585 nm wavelength was monitored continuously using a plate reader for 45 min. All points for experiments (n = 4) are shown in the graph with average shown as solid line. **c,** Sizing profile of EfpA^{EM} and SDS-PAGE gel. Numbers on the image correspond to labels on the peaks in the sizing profile. **d,** Sizing profile of purified EfpA^{EM} /Fab/Nb complex and SDS-PAGE gel. Peak 1 was used for cryo-EM grid preparation.

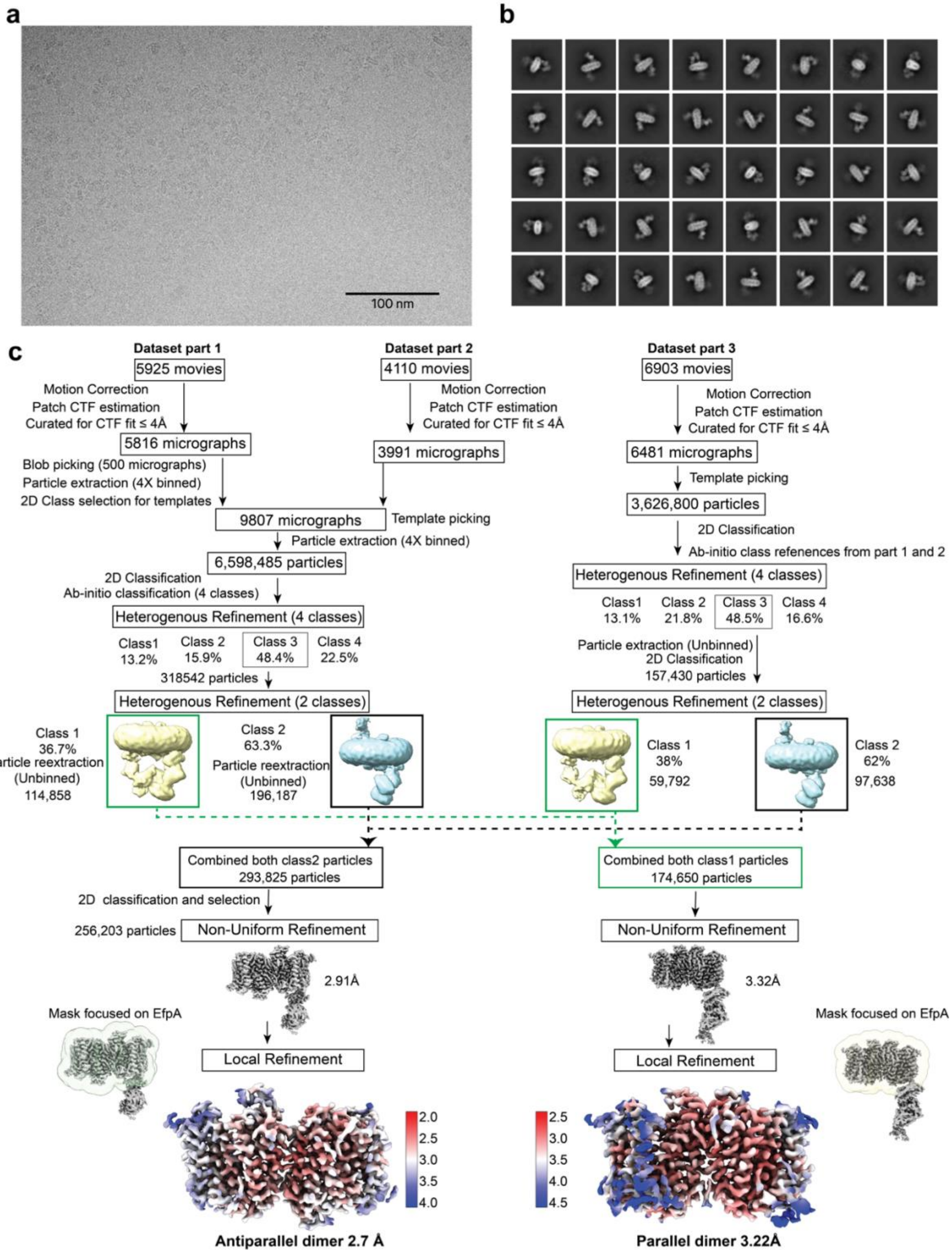

Supplementary Fig. 2 | EfpA apo cryo-EM data processing workflow. a, Representative micrograph image. **b,** Representative 2D classes form unbinned particles. **c,** Flowchart of 3D map generation and refinement in cryoSPARC. Map colored by local resolution of the final map calculated using cryoSPARC[1]. Left side shows the antiparallel dimer at global resolution 2.7 Å and right-side shows the parallel dimer at global resolution of 3.2 Å.

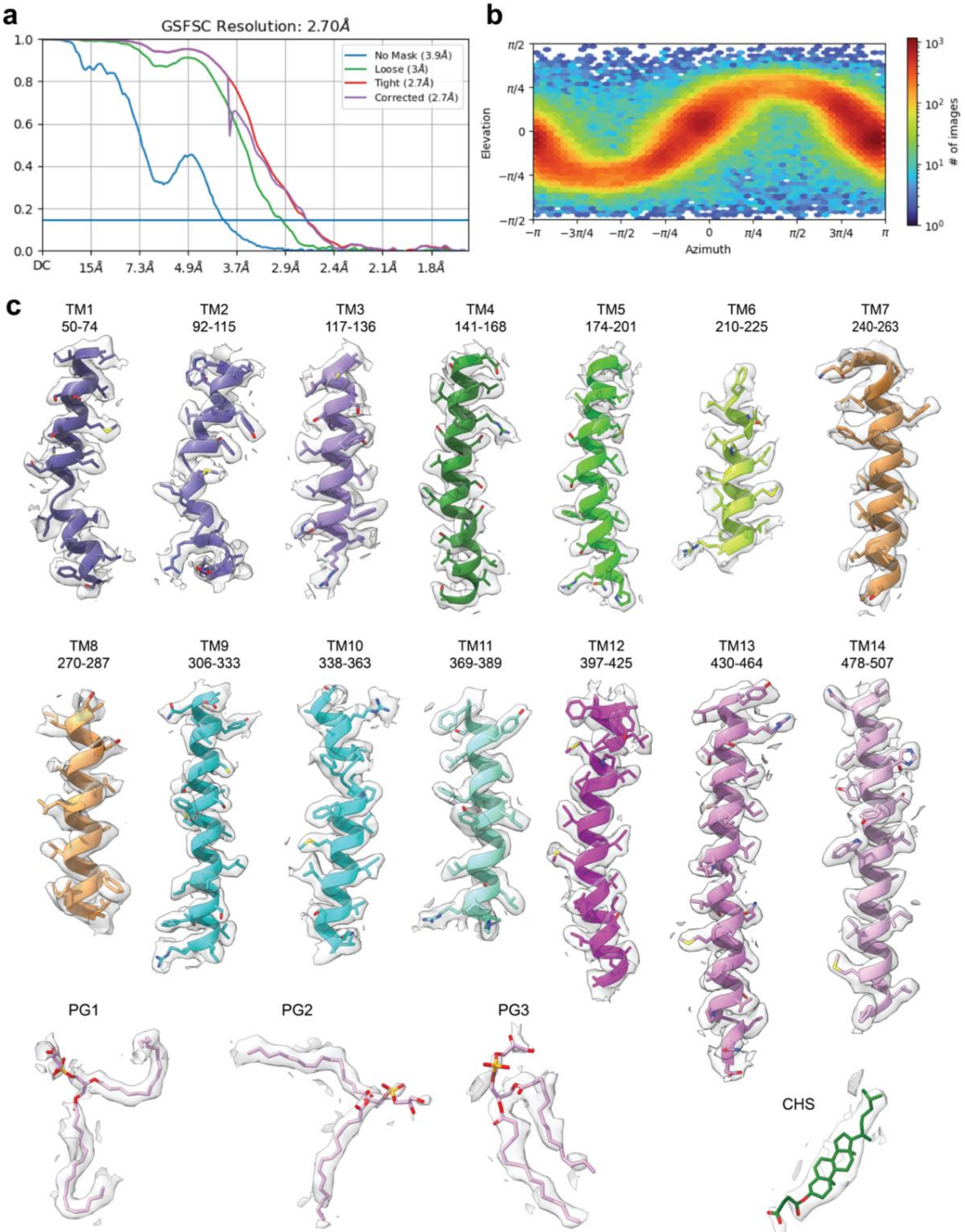

Supplementary Fig. 3 | Cryo-EM density of antiparallel dimer apo map. a, Fourier Shell Correlation (FSC) curve from final refinement showing a global resolution of 2.7 Å at a threshold of 0.143. **b,** Euler angle heatmaps of EfpA apo antiparallel structure. **c,** Final model, and corresponding map densities for EfpA monomer transmembrane helices and associated three PG and one CHS molecules.

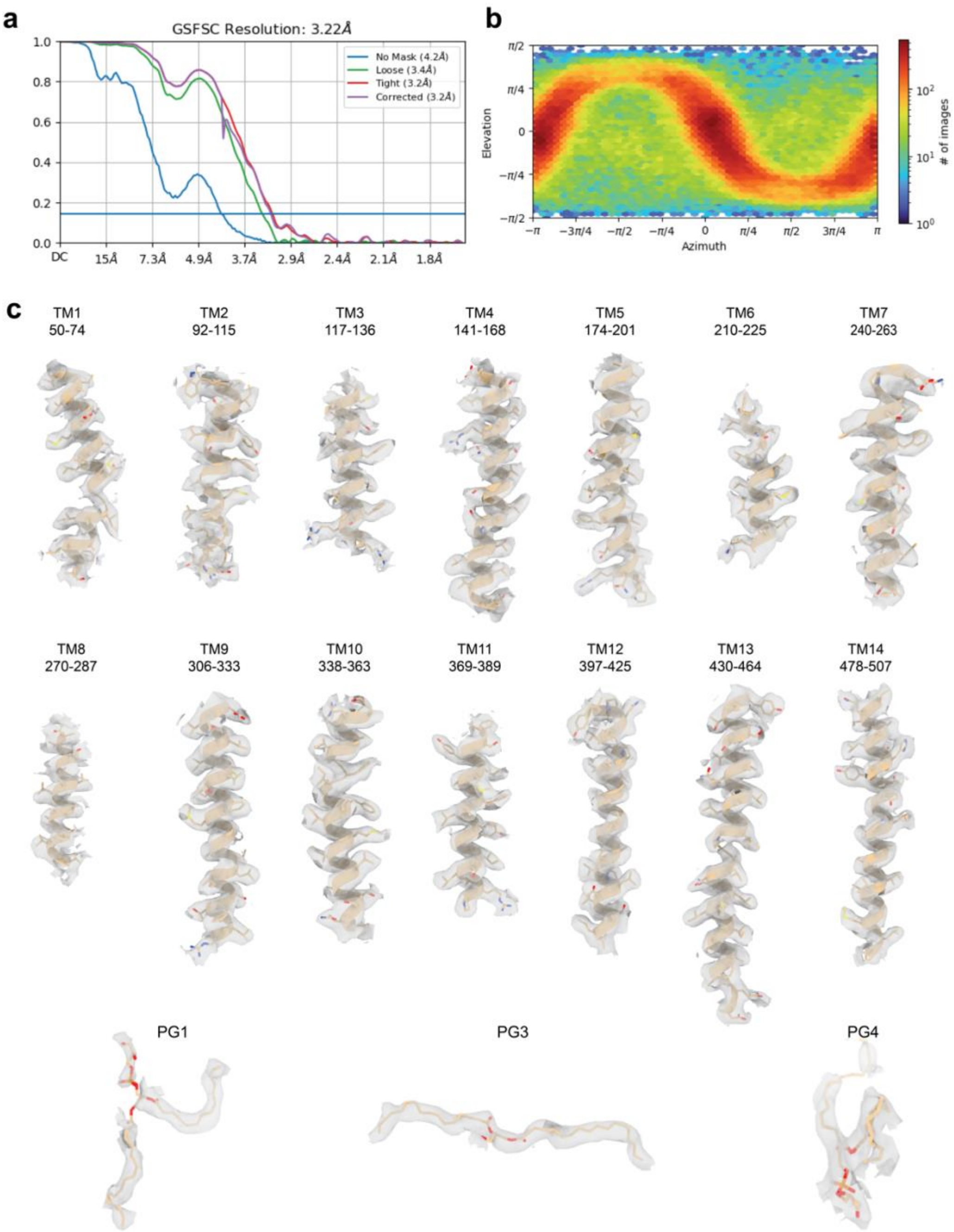

Supplementary Fig. 4 | Cryo-EM density of parallel dimer apo map. a, Fourier Shell Correlation (FSC) curve from final refinement showing a global resolution of 3.22 Å at a threshold of 0.143. **b,** Euler angle heatmaps of EfpA apo parallel structure. **c,** Final model, and corresponding map densities for EfpA monomer transmembrane helices and associated PG molecules.

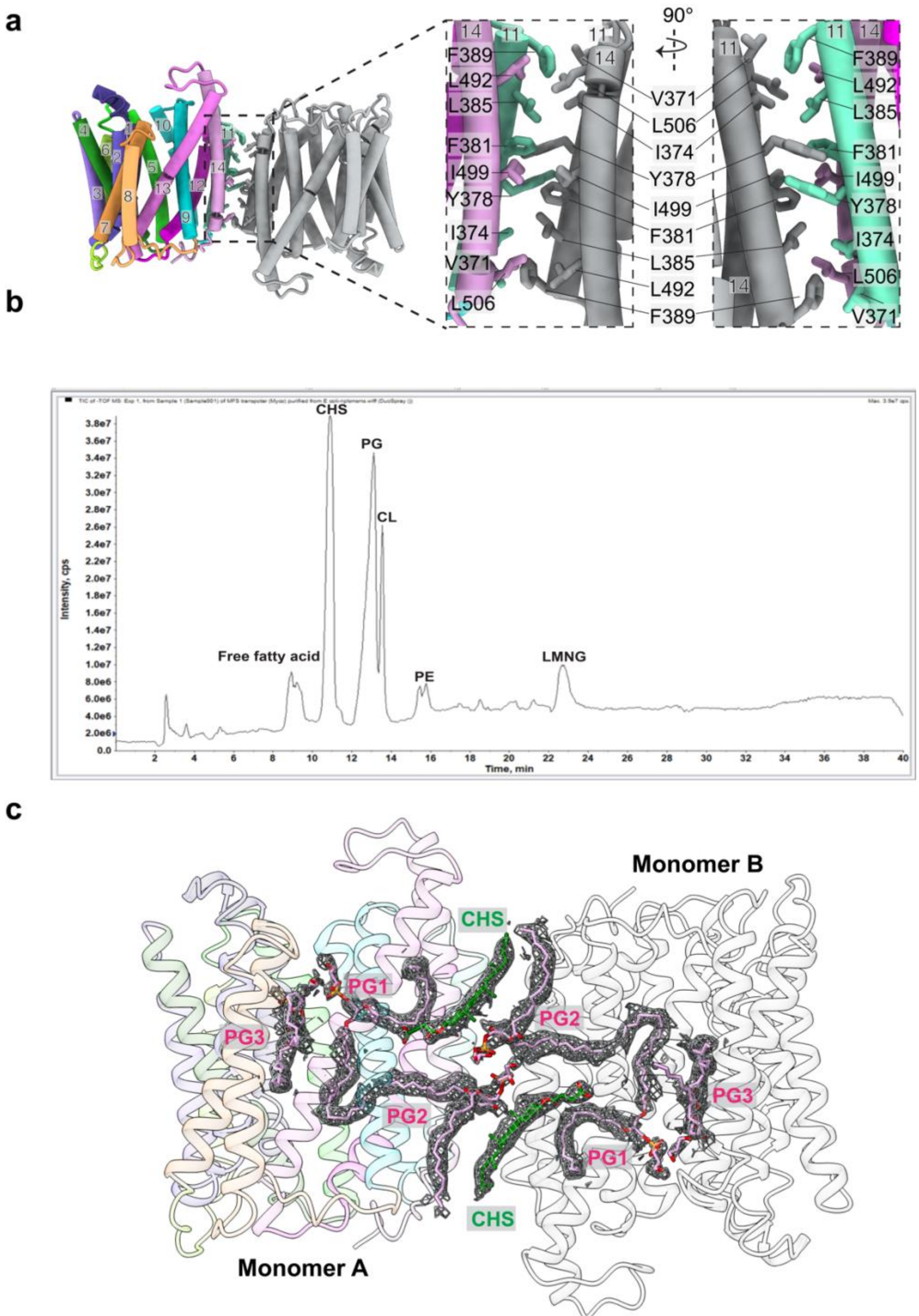

Supplementary Fig. 5 | Lipids in EfpA antiparallel dimer. a, Residues involved in the interface between two monomers in the antiparallel dimer. **b,** EfpA lipid identification by mass spectrometry**.** Total ion chromatogram of normal phase LC/MS in the negative ion mode. **c,** Cryo-EM density for the lipids PG1, PG2, PG3 and CHS.

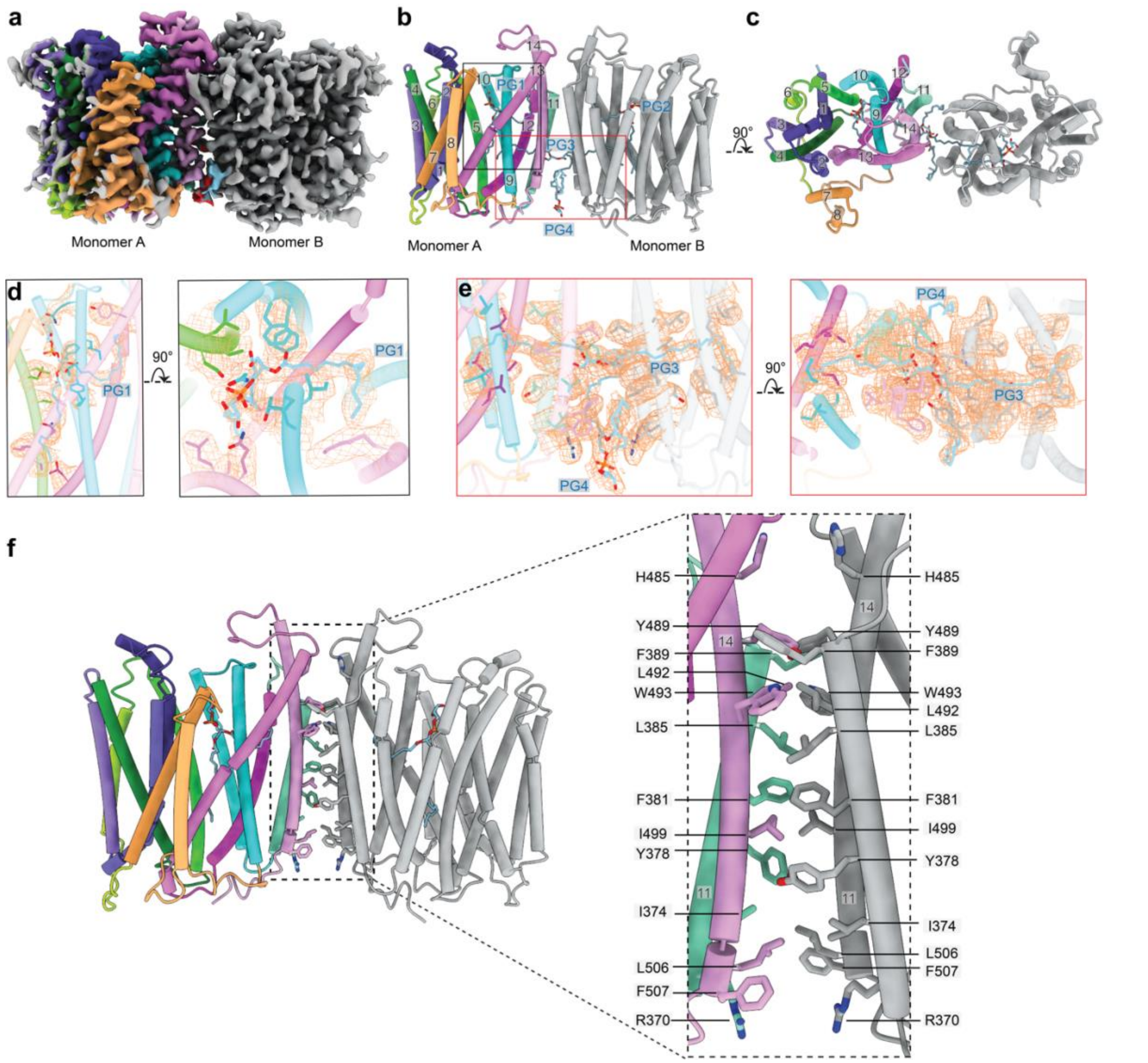

Supplementary Fig. 6 | Overall architecture of the parallel dimer a, Map for the parallel dimer. **b,** The structure of monomers A, B are schematized with lipids shown. **c,** top view as in b rotated by 90° down the two-fold axis between monomers. **d,** Density for the lipid PG1 from panel b and its interacting residues at a contour level at 0.3 at two different angles. **e,** Density for the lipid PG3 and 4 from panel b and its interacting residues at a contour level 0.3 from two different viewpoints. **f,** The interface between monomers showing residues involved in dimer formation.

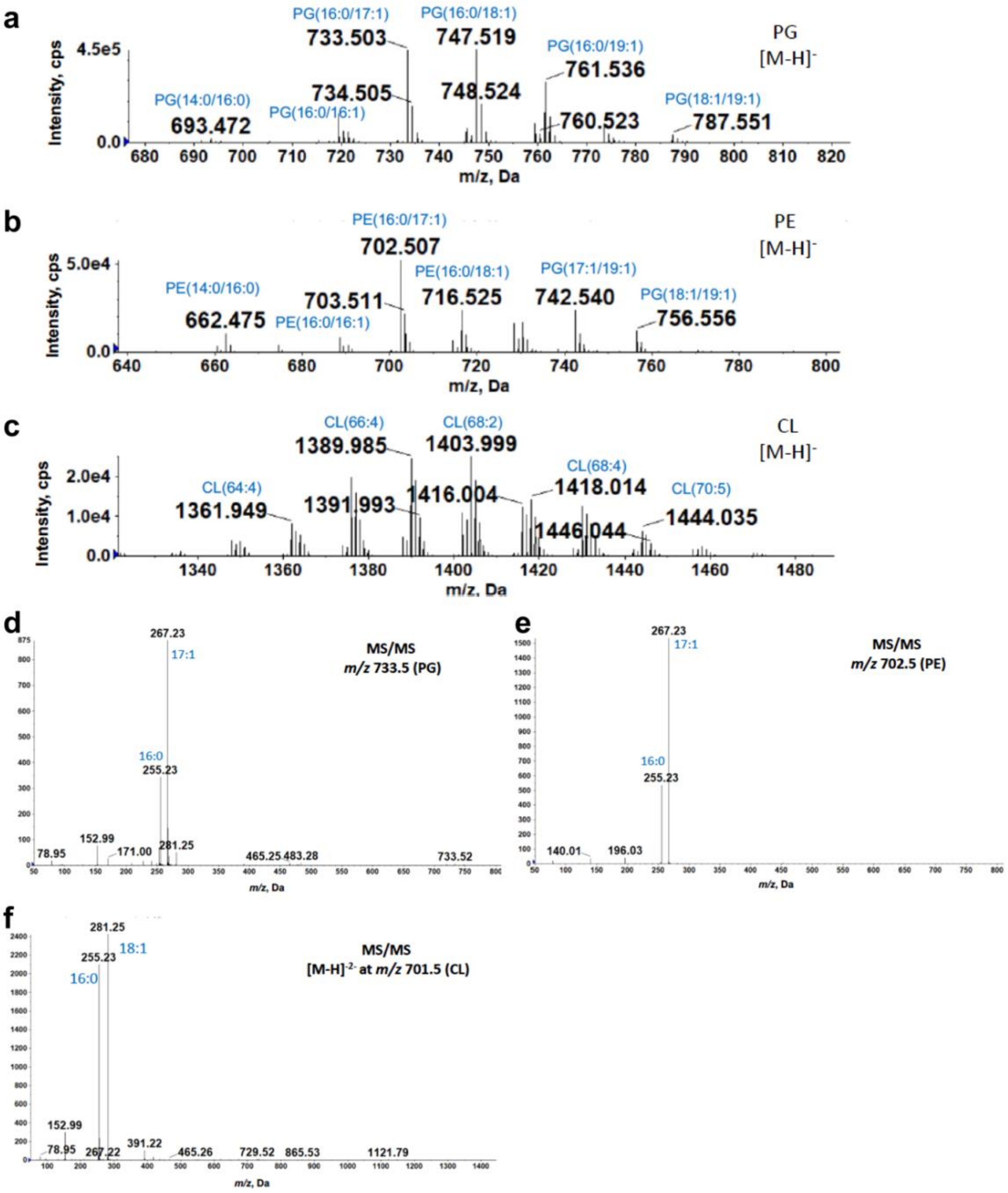

Supplementary Fig. 7 | LC/MS analysis of lipids bound to the recombinant EfpA protein purified from *E. coli*. Negative ion mass spectra of the $[M\text{-}H]^-$ ions of the molecular species of **a,** PG, **b,** PE and **c,** CL. In the parentheses, the numbers before the colon denote the carbon numbers of acyl chains, while the numbers after the colon denote the numbers of double bonds (or cyclopropanes). Representative negative ion MS/MS spectra of **d,** PG (16:0/17:1), **e,** PE (16:0/17:1) and **f,** CL (68:2). The acyl compositions are revealed by fatty acid anions shown in the product ion mass spectra. The MS analysis by the Q-Tof instrument was acquired in a scan-mode to detect all lipids. The assay was performed with one biological replicate (n=1).

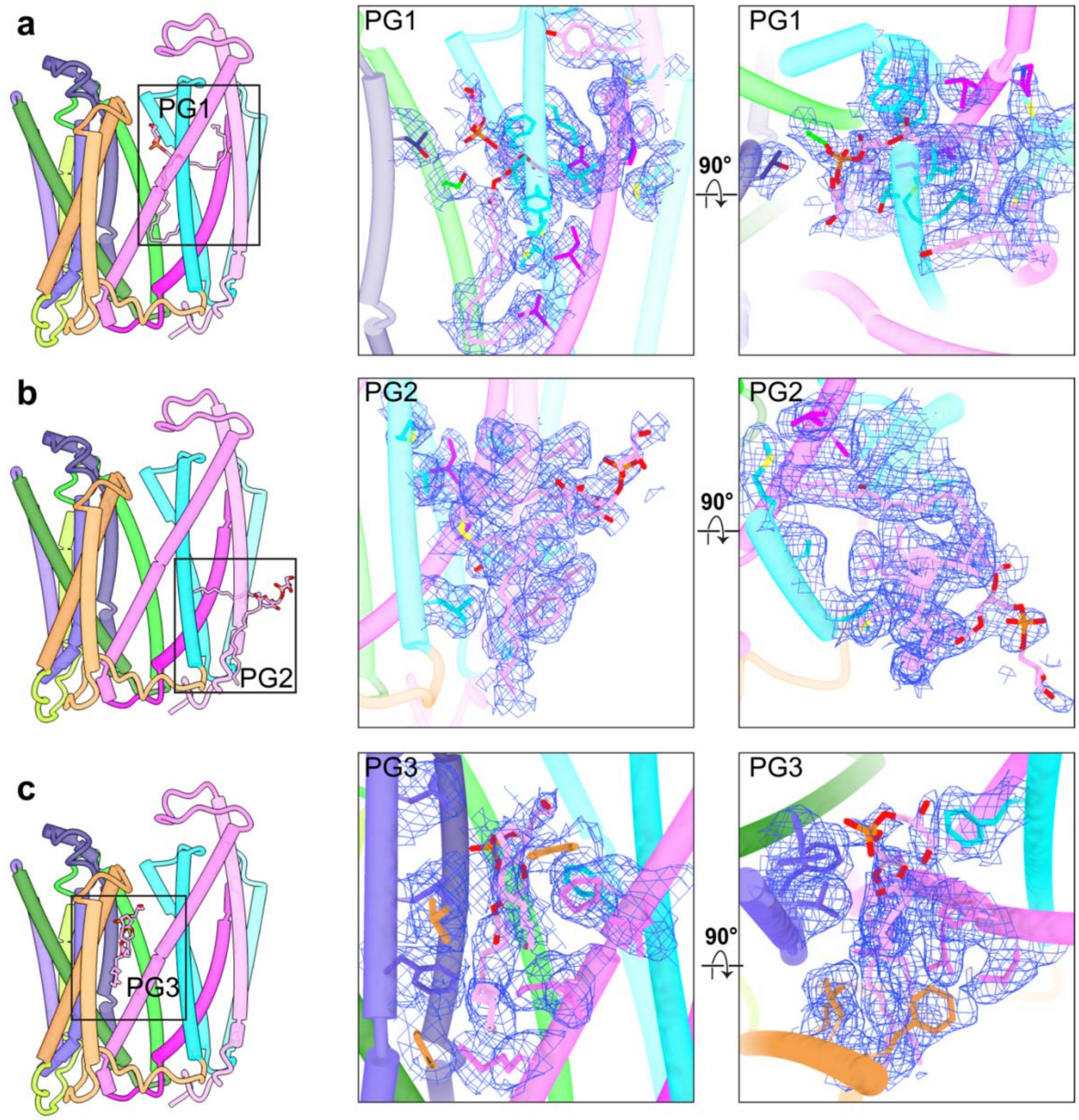

Supplementary Fig. 8 | Cryo-EM map density and model of PG molecules with its interacting residues. **a,** PG1**. b,** PG2**. c,** PG3 lipid molecules in EfpA monomer**.** Left most panel of **a, b, and c** shows EfpA with PG1, PG2 and PG3 molecules respectively. Middle and right panel respective PG molecules along with interacting sidechain of EfpA along with cryo-EM map density at a contour level at 0.3.

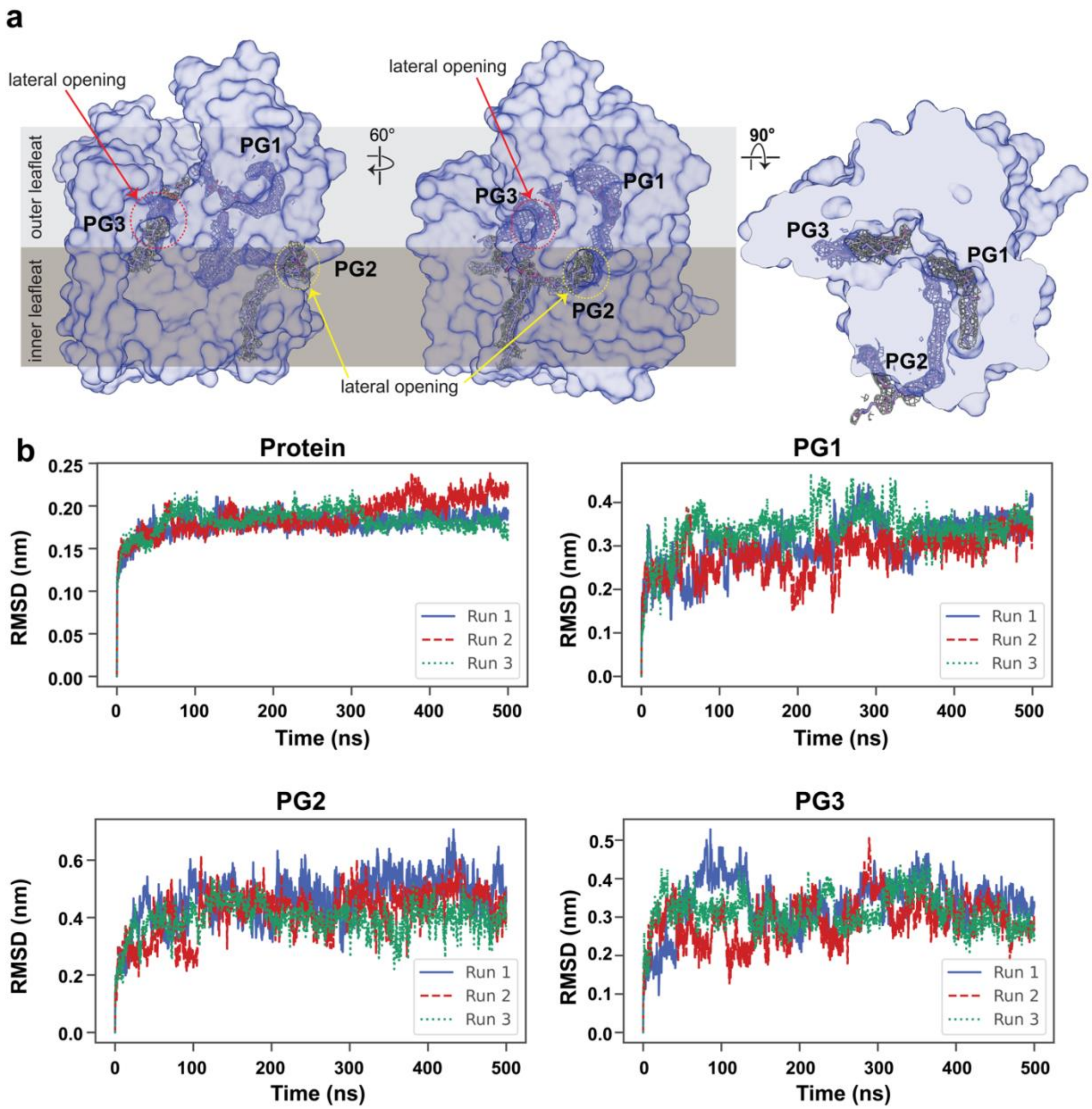

Supplementary Fig. 9 | Lipids molecules in EfpA monomer. a, Lateral openings in EfpA apo monomer A. Left panel showing lateral opening towards the outer leaflet in red dotted circle with PG3 molecules and density of it in cryo-EM map and lateral opening towards the inner leaflet in yellow dotted circle with PG2 molecules and density of it in cryo-EM map. Middle panel is rotation of right panel at 80°. Right panel left panel rotated by 90° showing the PG1, PG2 and PG3 binding positions. **b,** MD simulations illustrating the binding characteristics and durability of the lipid molecules (PG1, PG2 and PG3) when interacting with the protein EfpA. The results (top left) reveal that the protein is stable with an RMSD of < 2.5 Å (displayed through the protein and Cα curves above) and a consistently stable binding of the lipid molecules is observed over a duration of 500 ns. This is evident from the fact that the PG1 and PG3 (top and bottom right) molecules exhibit an RMSD of < 5 Å while the PG2 molecule (bottom left) exhibits a more perturbed motion with a higher RMSD of < 7 Å.

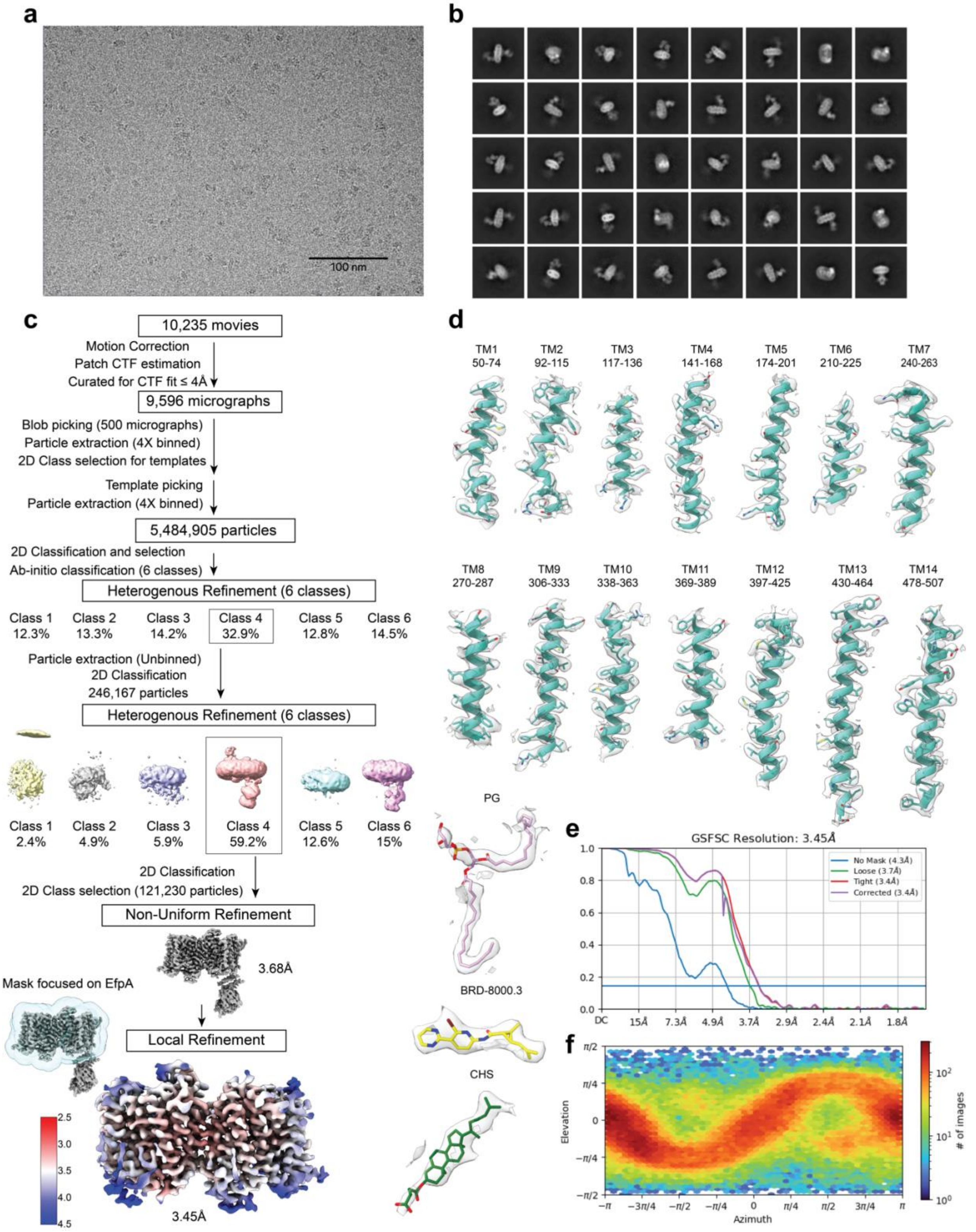

Supplementary Fig. 10 | Workflow of EfpA BRD-8000.3 bound data processing. a, Representative micrograph image. **b,** Representative 2D classes form unbinned particles extract. **c,** Flowchart of 3D-map generation and refinement in cryoSPARC[1]. Colored map for representation of local resolution of the final map calculated from cryoSPARC. **d,** Model and corresponding densities for EfpA monomer transmembrane helices and BRD-8000.3 and PG lipid molecule. **e,** Fourier Shell Correlation (FSC) curve from final refinement showing a global resolution of 3.45Å at a threshold of 0.143.**f,** Euler angle heatmaps of final map.

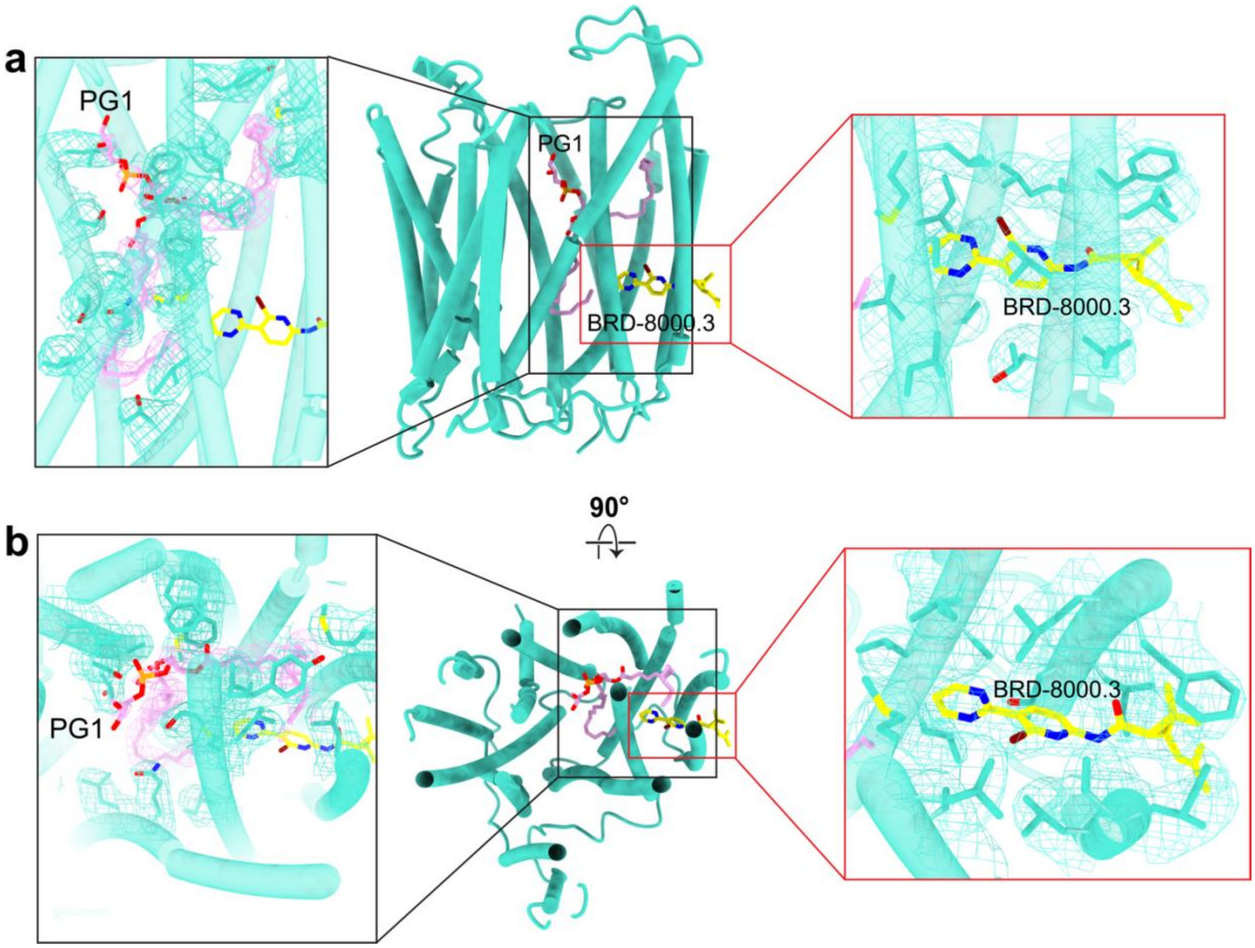

Supplementary Fig. 11 | EfpA bound BRD-8000.3 cryo-EM map and model quality for the ligand and interacting residues. a, Cryo-EM map density for the lipid PG1and its interacting residues (left) and BRD-8000.3 (right) at a contour level 0.5. **b,** top view as in a rotated by 90° from a. Section of lipid PG1 (left) and BRD-8000.3 (right) from panel b and its interacting residues cryo-EM map density at a contour level 0.5.

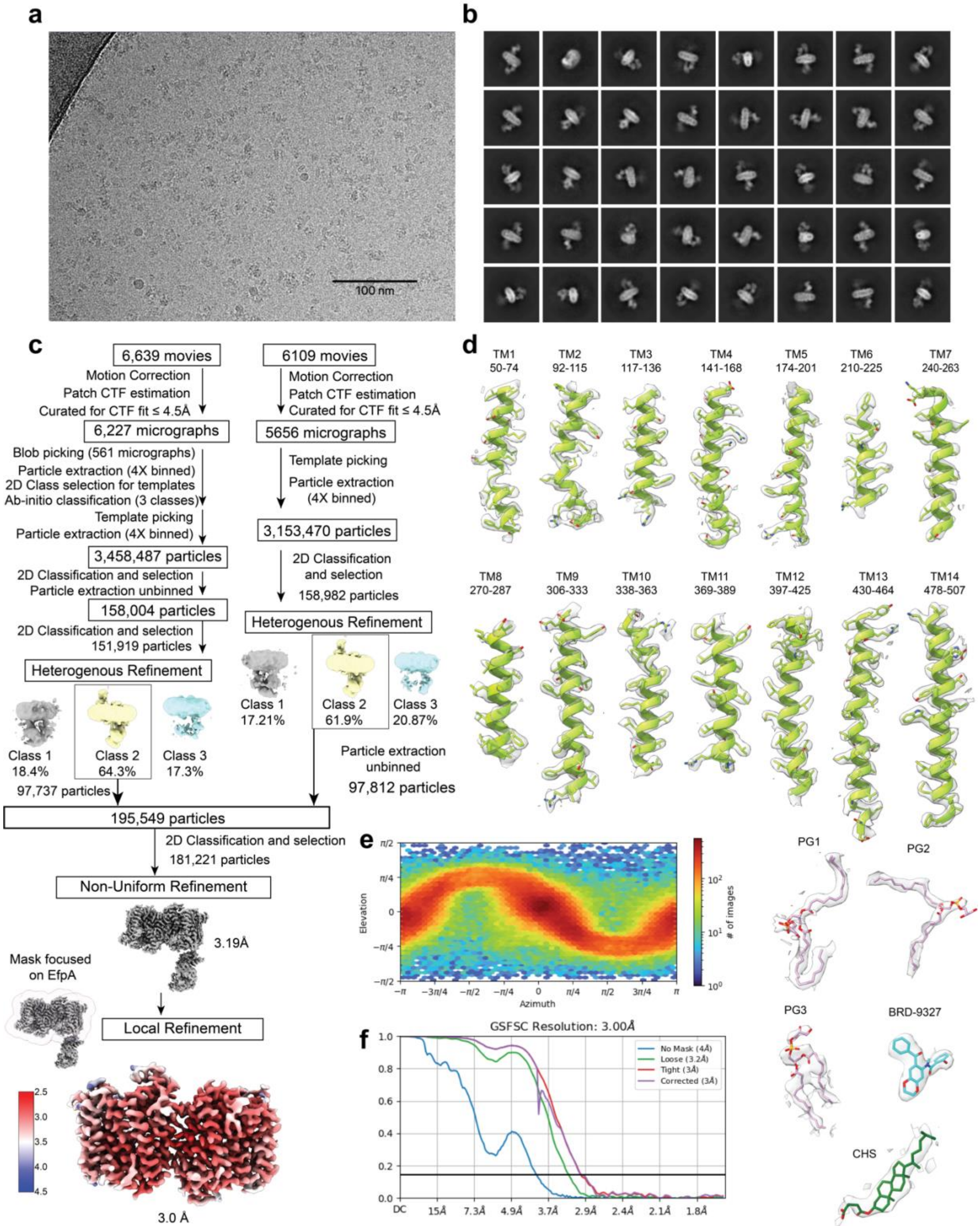

Supplementary Fig. 12 | Workflow of EfpA BRD-9327 bound cryo-EM data processing. a, Representative micrograph image. **b,** Representative 2D classes form unbinned particles extract. **c,** Flowchart of 3D-map generation and refinement in cryoSPARC[1]. Colored map for representation of local resolution of the final map calculated from cryoSPARC. **d,** Model and corresponding densities for EfpA monomer transmembrane helices and BRD-8000.3 and PG lipid molecule. **e,** Euler angle heatmaps of final map. **f,** Fourier Shell Correlation (FSC) curve from final refinement showing a global resolution of 3.0Å at a threshold of 0.143.

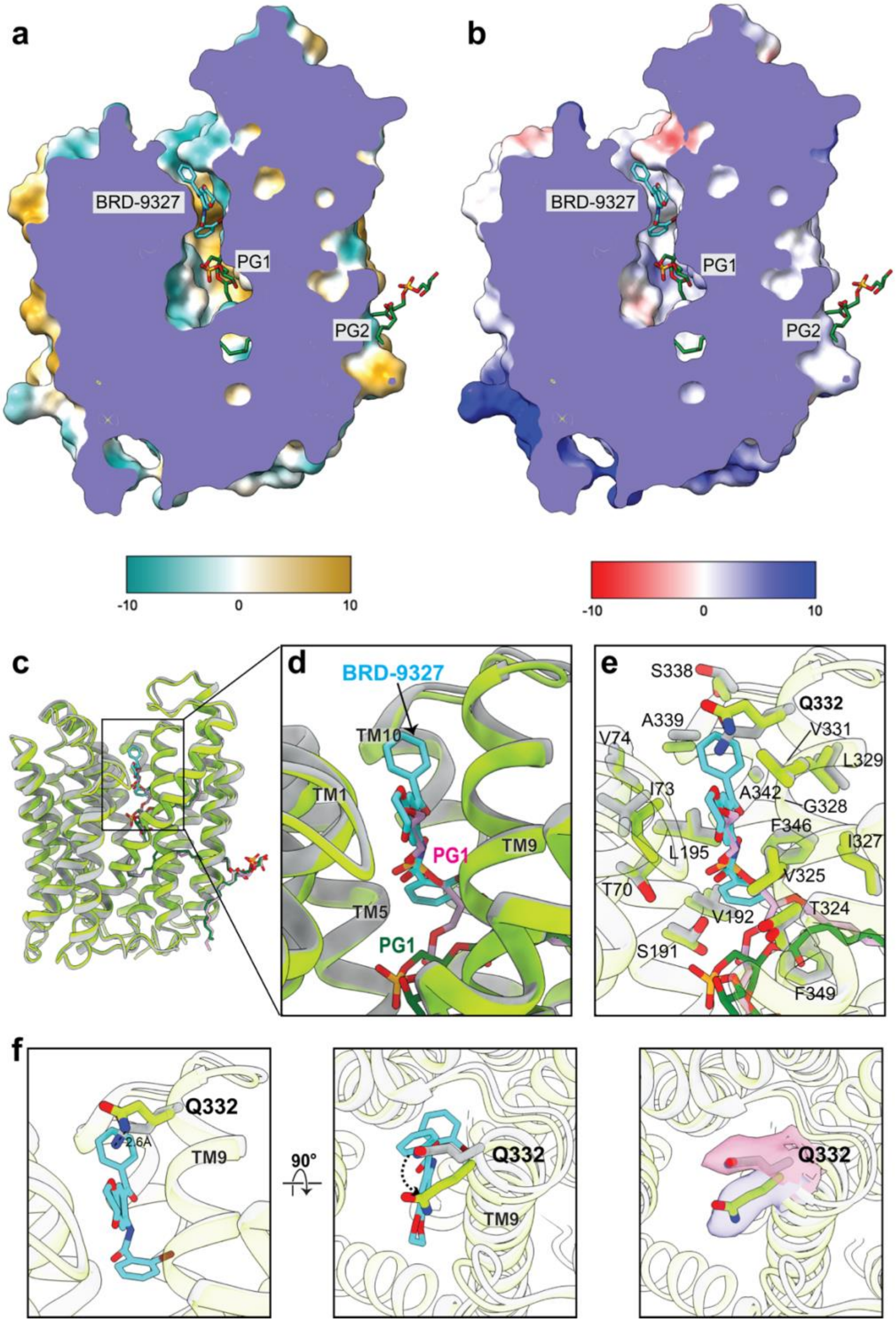

Supplementary Fig. 13 | Structural comparison of EfpA apo and BRD-9327 bound structures. a, section of the electrostatic surface of BRD-9327-bound EfpA showing the BRD-9327 binding site. **b**, as in a, colored according to hydrophobicity. **c,** superposition of BRD-9327 (green) onto the apo structure (gray) with lipids PG1 and PG2 (pink) as in the apo structure. BRD-9327 bound structure PG1 and PG2 shown in dark green and BRD-9327 (cyan) with nitrogen (blue), oxygen (red), and phosphate (orange). **d,** the BRD-9327 binding site and comparison with the apo structure, showing the overlap between PG1 of apo and BRD-9327. **e,** side chains of binding site shown in d. **f,** outward movement of the Q332 side chain in the BRD-9327 bound structure. Middle panel after rotating the structure 90° showing the top view. Right panel shows the EM density around the Q332 for BRD-9327 bound structure (blue) and apo structure (pink).

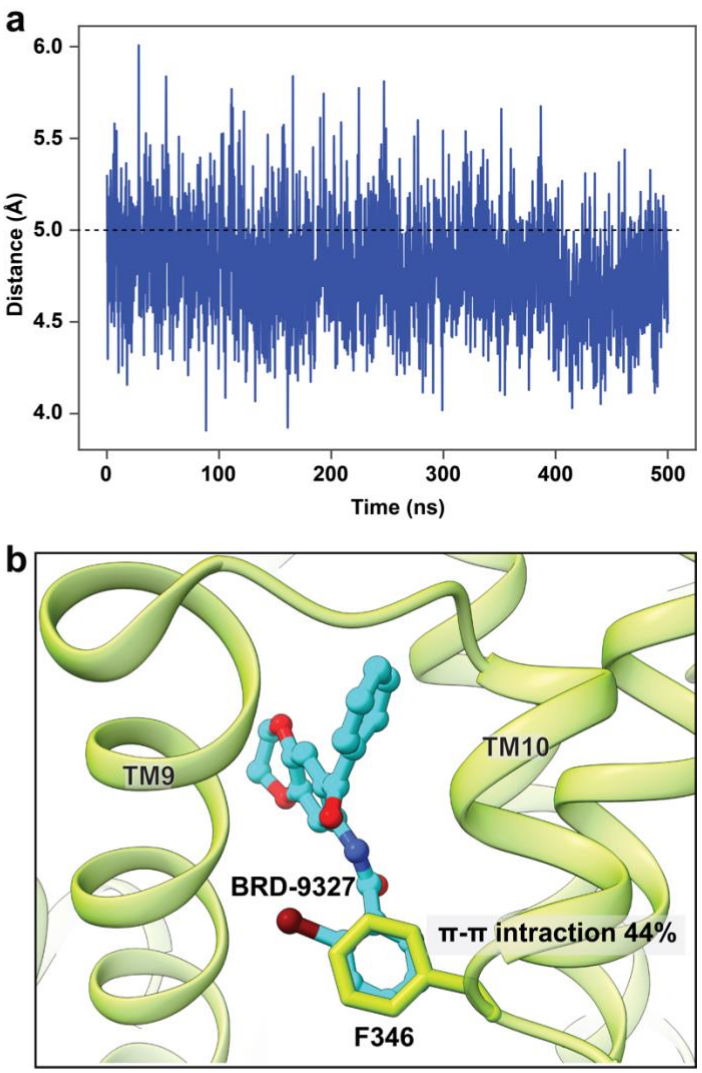

Supplementary Fig. 14 | Molecular dynamics simulations of BRD-9327 interaction with EfpA. a, distances between center of mas of BRD-9327 benzyl bromide ring and F346 side chain ring in 500 ns MD simulations. Distances less than 5 Å indicate π –π interactions (black dashed line). **b,** Representative snapshots (187 ns) for MD simulations of BRD-9327 binding to EfpA showing π–π interactions between BRD-9327 and $F346^{TM10}$. The π–π interactions between BRD-9327 and $F346^{TM10}$ were maintained for more ~ 44% throughout the simulation.

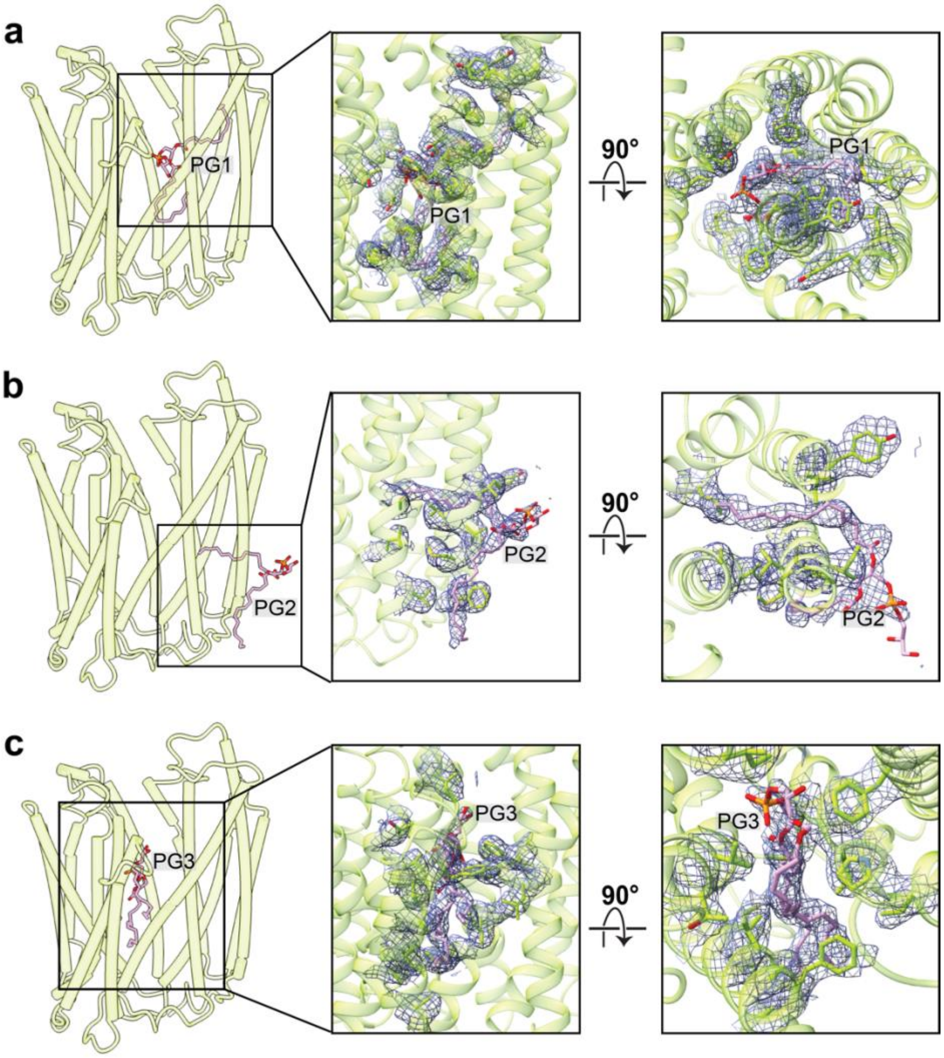

Supplementary Fig. 15 | EfpA bound BRD-9327 cryo-EM map and model quality for the PG molecules and interacting residues. a, Cryo-EM map density for the lipid PG1and its interacting residues. **b,** PG2 and its interacting residues. **c**, PG3 and its interacting residues at a contour level 0.509.

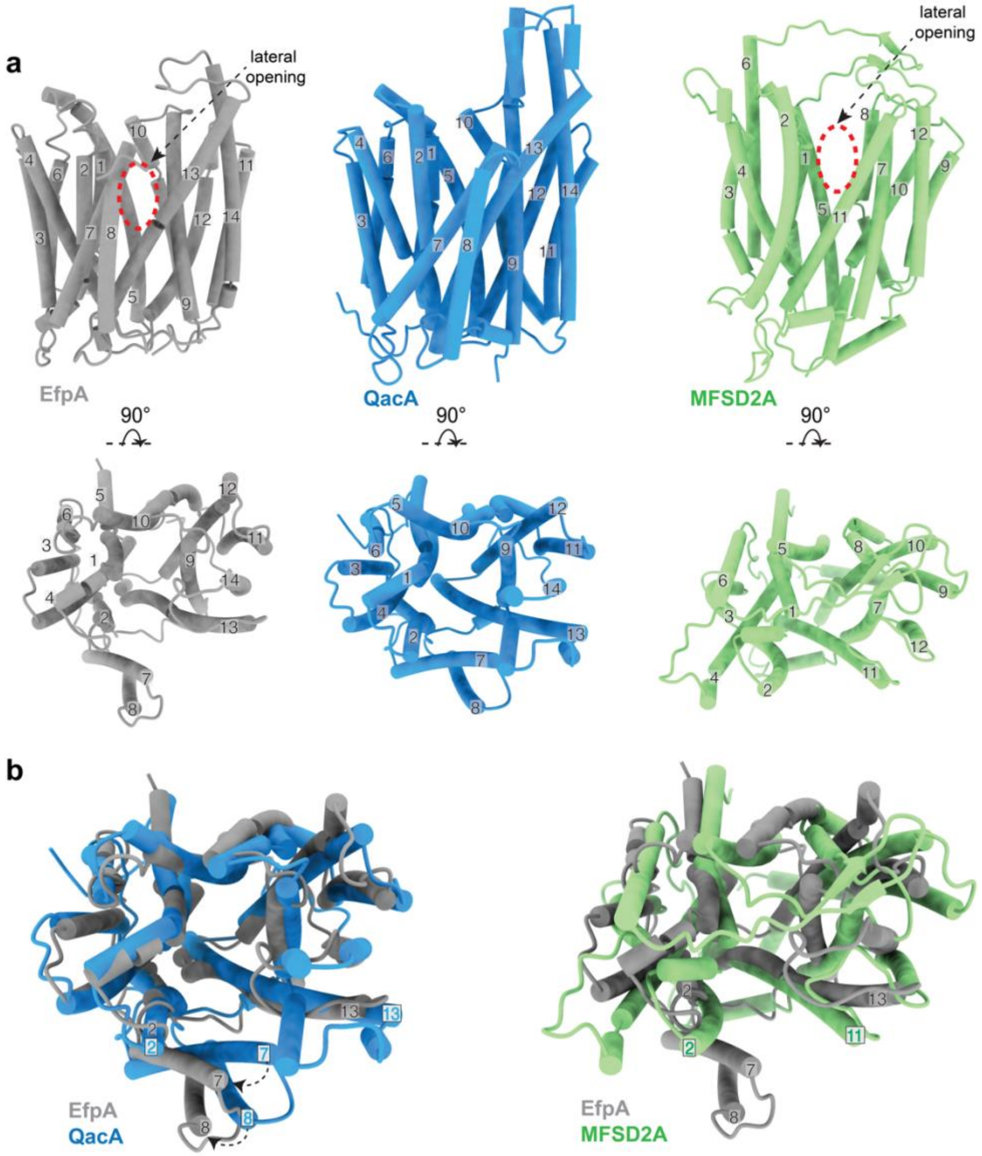

Supplementary Fig. 16 | Comparison of EfpA architecture with other DHA2 family transporters (QacA and NorC) and lipid transporter MFSD2A. a, Top panel, side view and bottom panel rotated by 90° to show the top view of the TM arrangement in EfpA, QacA (PDB: 7Y58)[2] and MFSD2A (PDB:7N98)[3] The extra linker between TM7 and TM8 in quaternary ammonium compound A family members (EfpA and QacA) lies next to the lateral opening between TM2 and TM13. EfpA TM7 and 8 arrange opposite towards TM2 in way that they don't block this lateral opening. In QacA TM7 and 8 present juxtaposed to space between TM2 and 13 and block the lateral opening. The absence of an extra linker domain in MFSD2A results in lateral opening similar to EfpA. **b,** Top view of overlapping structures of EfpA with QacA and MFSD2A highlighting the rearrangement of TM7 and 8.

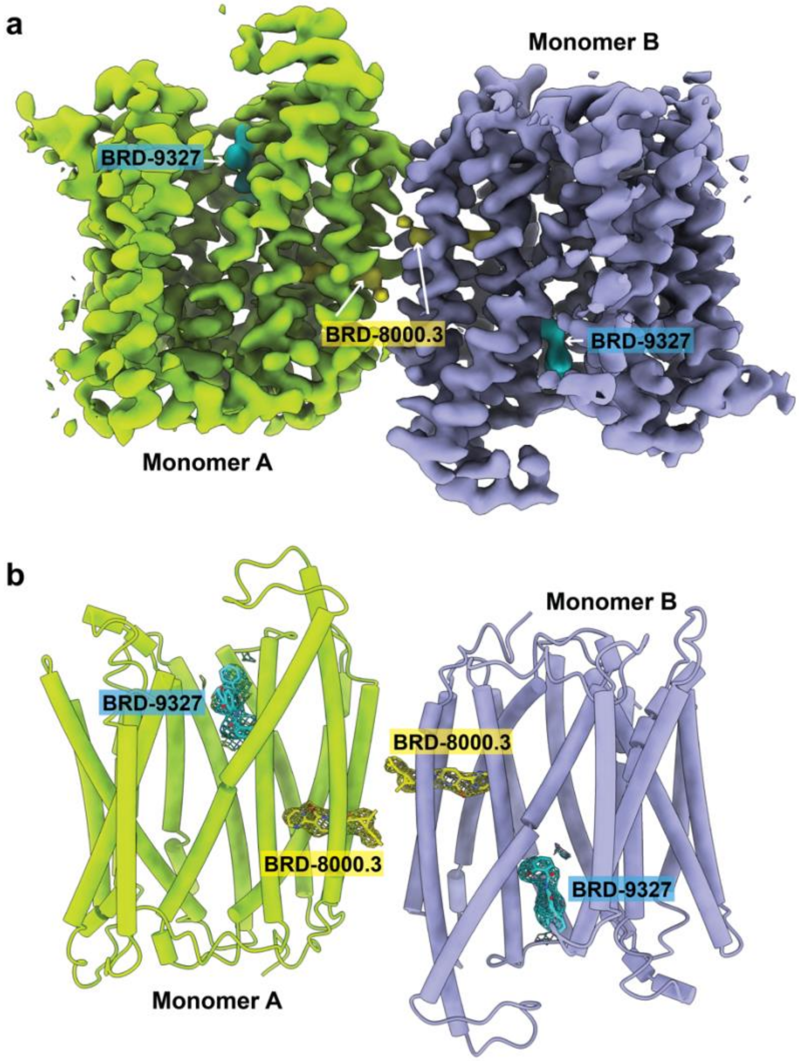

Supplementary Fig. 17 | Combinatorial inhibition of EfpA. a, cryo-EM map density of BRD-9327 and BRD-8000.3 bound $EfpA^{EM}$. Both monomers of EfpA shown as yellow-green and violet color respectively. Each monomer has one BRD-9327 molecule (aqua color) and one BRD-8000.3 (yellow color). **b,** Tertiary structure of $EfpA^{EM}$ monomer A with BRD-9327 (aqua) and BRD-8000.3 (yellow color) bound. Density for BRD-9327 (aqua) and BRD-8000.3 (yellow color) are shown as mesh. Color by atom nitrogen (blue), oxygen (red) and bromide (brown).

Supplementary Table 1				
**Structure**	EfpA (antiparallel dimer)	EfpA (parallel dimer)	BRD-8000.3 bound EfpA	BRD-9327 bound EfpA
**PDB**	**9BII**	**9BL7**	**9BIN**	**9BIQ**
**EMDB**	**44591**	**44651**	**44594**	**44598**
**Data collection / processing**				
Magnification	105,000	105,000	105,000	105,000
Voltage (kV)	300	300	300	300
Pixel size (Å)	0.835	0.835	0.4175	0.417
Pixel size after binning (Å)	0.835	0.835	0.835	0.834
Defocus range (μm)	-1 to -2	-1 to -2	-1 to -2	-1 to -2
Electron exposure (e/Å^2)	~45	~45	~45	~43
Symmetry imposed	C1	C1	C1	C1
Initial particles (No.)	10,225,285	10,225,285	5,484,905	6,611,957
Final particles (No.)	256,203	174,650	121,230	181,221
Map resolution (Å)	2.7	3.22	3.45	3.0
FSC threshold	0.143	0.143	0.143	0.143
Map resolution range (Å)	2.3 – 33.1	2.7 – 39.3	2.9 – 12.6	2.5 – 29.3
**Refinement**				
Initial model used	AF-P9WJY4-F1			
Model resolution (Å)	2.9	3.39	3.6	3.2
FSC threshold	0.5	0.5	0.5	0.5
Map sharpening B factor (Å^2)	88.2	91.7	119.2	101
Model composition				
Non-hydrogen atoms	7224	7059	7086	7283
Protein residues	940	940	942	942
Ligands	-	-	2	2
Lipids	6	4	2	6

B factors (Å^2)				
Protein	48.61	53.68	76.39	45.29
Ligand/Lipids	37.39	46.54	37.87	20
RMS deviations				
Bond lengths (Å)	0.006	0.003	0.005	0.008
Bond angles (°)	.607	0.535	0.558	0.690
Validation				
MolProbity score	1.20	1.49	1.53	1.33
Clashscore	2.68	4.76	5.46	4.14
Poor rotamers (%)	0	0	0	0
Ramachandran plot (%)				
Favored (%)	2.78	3.63	3.53	2.67
Allowed (%)	97.22	96.37	96.47	97.33
Disallowed (%)	0	0	0	0

Supplementary Table 1: Cryo-EM data collection, refinement and validation statistics

**Supplementary Table 2**	
**Primer**	**Sequence (5'-3')**
efpA_pUV_clone_F	GTTAATTAAGAAGGAGATATACATATGACGGCTCTCAACGACACAGAG
efpA_pUV_clone_R	GAATATTACAGCTCGCCGGCGTCGAT
V319A_F	TTGGCCGGCGGCGCCATGTTCAGCCTGA
V319A_R	TCAGGCTGAACATGGCGCCGCCGGCCAA
V319F_F	TTGGCCGGCGGCTTCATGTTCAGCCTGA
V319F_R	TCAGGCTGAACATGAAGCCGCCGGCCAA

Supplementary Table 2: Primer sequences

REFERENCES

1 Punjani, A., Rubinstein, J. L., Fleet, D. J. & Brubaker, M. A. cryoSPARC: algorithms for rapid unsupervised cryo-EM structure determination. *Nat Methods* **14**, 290-296, doi:10.1038/nmeth.4169 (2017).

2 Majumder, P. *et al.* Cryo-EM structure of antibacterial efflux transporter QacA from Staphylococcus aureus reveals a novel extracellular loop with allosteric role. *Embo J* **42**, e113418, doi:10.15252/embj.2023113418 (2023).

3 Wood, C. A. P. *et al.* Structure and mechanism of blood-brain-barrier lipid transporter MFSD2A. *Nature* **596**, 444-448, doi:10.1038/s41586-021-03782-y (2021).
