# Supplementary material for: Structure and inhibition mechanisms of Mycobacterium tuberculosis essential transporter efflux protein A
Source: Nat Commun. 2025 Apr 1;16:3139. doi: 10.1038/s41467-025-58133-6 (PMC11961569; doi:10.1038/s41467-025-58133-6)
Supplement: Supplementary file 2 — Description of Additional Supplementary Files [file 41467_2025_58133_MOESM2_ESM.pdf]

Description of Additional Supplementary Files

File Name: Supplementary Data 1

Description: Zip file of Simulation data.
