# Supplementary material for: Structure and inhibition mechanisms of Mycobacterium tuberculosis essential transporter efflux protein A
Source: Nat Commun. 2025 Apr 1;16:3139. doi: 10.1038/s41467-025-58133-6 (PMC11961569; doi:10.1038/s41467-025-58133-6)
Supplement: Supplementary file 4 — Reporting Summary [file 41467_2025_58133_MOESM4_ESM.pdf]

nature portfolio

Corresponding author(s): Robert M. Stroud

Last updated by author(s): 02/13/2025

Reporting Summary

Nature Portfolio wishes to improve the reproducibility of the work that we publish. This form provides structure for consistency and transparency in reporting. For further information on Nature Portfolio policies, see our Editorial Policies and the Editorial Policy Checklist.

Statistics

For all statistical analyses, confirm that the following items are present in the figure legend, table legend, main text, or Methods section.

n/a	Confirmed	
☐	☒	The exact sample size (*n*) for each experimental group/condition, given as a discrete number and unit of measurement
☐	☒	A statement on whether measurements were taken from distinct samples or whether the same sample was measured repeatedly
☒	☐	The statistical test(s) used AND whether they are one- or two-sided<br>*Only common tests should be described solely by name; describe more complex techniques in the Methods section.*
☒	☐	A description of all covariates tested
☒	☐	A description of any assumptions or corrections, such as tests of normality and adjustment for multiple comparisons
☐	☒	A full description of the statistical parameters including central tendency (e.g. means) or other basic estimates (e.g. regression coefficient) AND variation (e.g. standard deviation) or associated estimates of uncertainty (e.g. confidence intervals)
☒	☐	For null hypothesis testing, the test statistic (e.g. *F*, *t*, *r*) with confidence intervals, effect sizes, degrees of freedom and *P* value noted<br>*Give P values as exact values whenever suitable.*
☒	☐	For Bayesian analysis, information on the choice of priors and Markov chain Monte Carlo settings
☒	☐	For hierarchical and complex designs, identification of the appropriate level for tests and full reporting of outcomes
☒	☐	Estimates of effect sizes (e.g. Cohen's *d*, Pearson's *r*), indicating how they were calculated

Our web collection on statistics for biologists contains articles on many of the points above.

Software and code

Policy information about availability of computer code

Data collection	SerialEM v4.1 beta was used for automated EM-data collection.
Data analysis	cryoSPARC v4.2.1 , MotionCor2v1.4.1, COOT 0.9, Phenix 1.21, UCSF-ChimeraX1.4, UCSF-ChimeraX1.1, MOLEonline server, AlphaFold2 and Graphpad prism 9, TF 1.5, CHARMM-GUI, TopSpin 3.6

For manuscripts utilizing custom algorithms or software that are central to the research but not yet described in published literature, software must be made available to editors and reviewers. We strongly encourage code deposition in a community repository (e.g. GitHub). See the Nature Portfolio guidelines for submitting code & software for further information.

Data

Policy information about availability of data

All manuscripts must include a data availability statement. This statement should provide the following information, where applicable:

- Accession codes, unique identifiers, or web links for publicly available datasets
- A description of any restrictions on data availability
- For clinical datasets or third party data, please ensure that the statement adheres to our policy

The atomic coordinates for four structures of EfpA apo antiparallel dimer, parallel dimer, BRD-9327 bound and BRD-8000.3 bound have been deposited in the Protein Data Bank with the accession codes PDB ID 9BII (https://doi.org/10.2210/pdb9BII/pdb) (EfpA antiparallel dimer), 9BL7 (https://doi.org/10.2210/pdb9BL7/pdb) (EfpA parallel dimer), 9BIQ (https://doi.org/10.2210/pdb9BIQ/pdb) (BRD-9327 bound EfpA) and 9BIN (https://doi.org/10.2210/pdb9BIN/pdb) (BRD-8000.3 bound EfpA) respectively. The corresponding maps have been deposited in the Electron Microscopy Data Bank with the accession codes EMD-44591(https://

www.emdataresource.org/EMD-44591) (EfpA antiparallel dimer), EMD-44651(https://www.emdataresource.org/EMD-44651) (EfpA parallel dimer), EMD-44598 (https://www.emdataresource.org/EMD-44598) (BRD-9327 bound EfpA) and EMD-44594 (https://www.emdataresource.org/EMD-44594) (BRD-8000.3 bound EfpA).

Research involving human participants, their data, or biological material

Policy information about studies with human participants or human data. See also policy information about sex, gender (identity/presentation), and sexual orientation and race, ethnicity and racism.

Reporting on sex and gender	Not applicable
Reporting on race, ethnicity, or other socially relevant groupings	Not applicable
Population characteristics	Not applicable
Recruitment	Not applicable
Ethics oversight	Not applicable

Note that full information on the approval of the study protocol must also be provided in the manuscript.

Field-specific reporting

Please select the one below that is the best fit for your research. If you are not sure, read the appropriate sections before making your selection.

- [x] Life sciences
- [ ] Behavioural & social sciences
- [ ] Ecological, evolutionary & environmental sciences

For a reference copy of the document with all sections, see nature.com/documents/nr-reporting-summary-flat.pdf

Life sciences study design

All studies must disclose on these points even when the disclosure is negative.

Sample size	Sample size calculation not performed. EM- data collected based on best practice used in the field. Transport assay was performed as technical quadruplicate . All MIC assays are done at least in triplicate. lipid identification analysis is performed as one biological replicate.
Data exclusions	All raw cryo-EM data were processed through the cryoSPARC v4.2.1 a pipelines. Micrographs were excluded based on poor resolution estimates using CTFFIND4 in cryoSPARC which often correlated with heavy ice contamination. Particle selection was performed following 2D and 3D classification in cryoSPARC v4.2.1 and judging by predicted resolution and visual inspection of classes.
Replication	All experiments were performed at least in triplicate otherwise mentioned in figure legends. All attempts were successful and gave consistent results.
Randomization	Particles were randomized into two half sets following the standard automated cryoSPARC protocol.
Blinding	Researchers were not blinded to group allocation. All internal assignment to different classes were performed internally by the reconstruction software (ccryoSPARCv4.2.1).

Reporting for specific materials, systems and methods

We require information from authors about some types of materials, experimental systems and methods used in many studies. Here, indicate whether each material, system or method listed is relevant to your study. If you are not sure if a list item applies to your research, read the appropriate section before selecting a response.

Materials & experimental systems

n/a	Involved in the study	
x		Antibodies
x		Eukaryotic cell lines
x		Palaeontology and archaeology
x		Animals and other organisms
x		Clinical data
x		Dual use research of concern
x		Plants

Methods

n/a	Involved in the study	
x		ChIP-seq
x		Flow cytometry
x		MRI-based neuroimaging

Plants

Seed stocks	N/A
Novel plant genotypes	N/A
Authentication	N/A
